# Spatial heterogeneity of neighborhood-level water and sanitation access in informal urban settlements: A cross-sectional case study in Beira, Mozambique

**DOI:** 10.1371/journal.pwat.0000022

**Published:** 2022-06-09

**Authors:** Courtney Victor, Denisse Vega Ocasio, Zaida A. Cumbe, Joshua V. Garn, Sydney Hubbard, Magalhaes Mangamela, Sandy McGunegill, Rassul Nalá, Jedidiah S. Snyder, Karen Levy, Matthew C. Freeman

**Affiliations:** 1 Gangarosa Department of Environmental Health, Emory University, Atlanta, Georgia, United States of America; 2 WEConsult, Maputo, Mozambique; 3 School of Community Health Sciences, University of Nevada, Reno, Nevada, United States of America; 4 AURA – Autoridade Reguladora de Água, Former Executive Secretary of the Water Regulation Council (CRA), (AURA, IP), Water Regulatory Authority, Public Institute, Maputo, Mozambique; 5 INS – Instituto Nacional de Saúde, Ministério de Saúde, Maputo, República de Moçambique; 6 Department of Environmental and Occupational Health Sciences, School of Public Health, University of Washington, Seattle, Washington, United States of America

## Abstract

Rapid urbanization, resulting in population growth within informal settlements, has worsened exclusion and inequality in access to water and sanitation (WASH) services in the poorest and most marginalized communities. In this study, we describe the heterogeneity in water service satisfaction and WASH access in low-income, peri-urban neighborhoods of Beira, Mozambique, and examine whether this heterogeneity can be explained by distance to water distribution mains. Using spatial statistics and regression analyses, we identify spatial heterogeneity in household WASH access, as well as consumer-reported satisfaction with water services (services, pressure, quality, and sufficient quantity). We find that as distance from the water main increased, both access to an improved water source at the household and satisfaction with water pressure decreases, and water supply intermittency increases, controlling for household density and socioeconomic status. The odds of a household having access to a water source at the household or on the compound decreases with every 100-meter increase in distance from a water main pipe (odds ratio [OR] 0.87, 95% confidence interval [CI]: 0.82, 0.92). Satisfaction with water services also decreases with every 100-meter increase in distance from a water main pipe (OR: 0.80; 95% CI: 0.69, 0.94). Days of availability in the past week decreases by a factor of 0.22 for every 100-meter increase in distance from the water main (95% CI: −0.29, −0.15). Findings from this study highlight the unequal household access to water and sanitation in urban informal settlements, even within low-income neighborhoods. Describing this heterogeneity of access to water services, sanitation, and satisfaction—and the factors influencing them—can inform stakeholders and guide the development of infrastructural solutions to reduce water access inequities within urban settings.

## Introduction

Rapid urbanization in low- and middle-income countries (LMICs) has brought challenges and urgency in the provision of access to improved water and sanitation services [1]. In sub-Saharan Africa, nearly 60% of people living in cities reside in informal settlements, defined as urban areas where residents lack access to basic public services, goods and amenities, and formal and secure tenure [2]. Population growth within informal settlements has overstretched existing water supply and sewerage networks. While the number of people living without safely managed services decreased overall between 2015 and 2020, it increased by 32 million in urban areas [1]. The challenge of providing services to a larger population has been exacerbated by structural challenges and weaknesses in water governance [3], leading to a lack of secure tenure and water service provision. The mismatch in investment, expansion, and maintenance in urban water and sanitation services has resulted in delays in the expansion of services to informal settlement areas, as well as reduced operational sustainability of these services [4–7].

As of 2018, 4.2 billion people were living in urban areas globally; of those, about 300 million were children [8, 9], and poor access to basic water, sanitation and hygiene (WASH) remains a global risk factor for exposure to fecal pathogens [10, 11]. Diarrheal diseases accounted for almost 1.6 million deaths worldwide in 2017, and they were the second leading cause of mortality in children under the age of five [12, 13]. Lack of WASH access contributes to outbreaks of waterborne and respiratory diseases, including COVID-19, as well as malnutrition and impaired educational outcomes and social and economic development [14–18]. These conditions are exacerbated in children living in urban, informal settlements, who experience higher morbidity and mortality as a result of limited access to water and sanitation facilities [19–22].

Access to water and sanitation services within urban informal settlements is typically poor, but not uniformly so. While limited access to water and sanitation services is more pronounced in informal and urban settlements [23, 24], there is limited data on heterogeneity *within* urban informal settlements, and a lack of analysis on what drives these underlying inequities. Among the factors known to contribute to this problem are challenges with distribution network engineering in informal neighborhoods, issues of housing and network expansion planning, and failure of public policy to provide satisfactory solutions to both address and ensure access to safe and continuous water supply [23–25]. While progress has been made in addressing water and sanitation inequities, global measures of coverage overestimate those with reliable, high-quality services [11, 23, 26]. Further, measures of coverage do not capture factors such as quality or equitable service delivery. Equitable access to safe water and sanitation systems is achieved as long as the principles of operational sustainability are upheld [27]. These include both the functionality of the systems themselves and the household’s experience of quality services (e.g., satisfaction with water quality and service delivery) over time [27].

Significant disparities in WASH access within countries exist, and are commonly reported, between the income levels and urban-rural living conditions [11]; however, smaller scale (i.e., regional or city-wide) measures of inequity is not well understood [11, 28–31]. A few studies have employed spatial tools to investigate heterogeneity in access at a high spatial resolution (i.e., within a city). In Nepal, more heterogeneity of access was found within provinces than between provinces, particularly within urban provinces [32]. In sub-Saharan Africa, estimated district-level WASH access within at least 10 countries ranged from 25% to more than 75% [33]. These findings highlight how national or regional WASH-coverage statistics can mask local inequities in WASH access. Understanding local variability in water and sanitation access, and the factors influencing heterogeneous access in informal settlements, could support more targeted, equitable, and appropriate improvements to expand reliable, high-quality coverage and access.

Relative to other African countries of similar income, Mozambique has experienced fewer and unequal improvements in access to basic water and sanitation services since 2010. Access to at least basic water sources in Mozambique was approximately 63% in 2020 [11]. In the same year, access to at least basic sanitation services was around 37% in Mozambique which was lower than Nigeria (43%), and Rwanda (69%) [11]. Water and sanitation coverage rates in Mozambique are higher in urban areas than rural areas (61% vs. 23% for at least basic sanitation and 65% vs. 14% for accessible water on premises), yet access to at least basic facilities is unevenly distributed across the country [11, 31]. It is one of 15 countries with a gap in subnational basic sanitation coverage of greater than 50%, and the ratio for basic drinking water coverage comparing the richest wealth quintiles to the poorest wealth quintiles was 1.8 in 2019 [11]. The slow and unequal increase in facilities in urban areas of Mozambique has been attributed to limited resources and funding to maintain or improve current infrastructure, limited emphasis on service and water quality, and challenges related to informal settlements infrastructures [11, 31, 34].

In Mozambique, households typically access water through three different mechanisms: 1) private household connections, 2) public standpipes, 3) neighbors’ taps [35–37]. Zuin et al. found that individuals who have a private household connection in Maputo, the urban capital of Mozambique, tend to be wealthier and spend a smaller percentage of their income on water compared to individuals who utilize public standpipes and neighbors’ taps [35, 37]. Individuals with a household connection report being most satisfied with their water service and report the most hours per day of water availability [35, 37]. However, establishing a household connection to the public water utility is cost-prohibitive, despite attempts by the water utility to reduce the fee for connection [36]. Even when there are no differences in microbiological water quality between water sources, as reported by Zuin et al. [38], there are other important limitations in using public standpipes and purchasing water from neighbors. While public standpipes are a less expensive alternative to increase safe water access [5], consumers who use a public standpipe pay a higher price per unit volume and experience greater time expenditures in water collection [37]. Additionally, previous studies have found a limited impact on health from shared water sources, such as standpipes, compared to unimproved water sources [39, 40]. Individuals who purchase water from their neighbors’ taps have reduced time expended on collection, but report the least amount of availability per day, pay more per unit volume than individuals with household connections, and describe feelings of humiliation about relying on their neighbors for their water supply [37]. Findings from this previous body of work in informal settlements in urban Maputo highlight important benefits associated with a household connection to an improved water supply. Details on who has access to water and sanitation services within informal settlements in Mozambique, and drivers of that access, can help identify optimal strategies for increasing service provision for all.

The purpose of this study is to describe the heterogeneity in household access to improved water and sanitation services in low-income, urban neighborhoods of Beira, Mozambique. We report on data that facilitate understanding of the drivers of variability of access to improved water and sanitation facilities, even within urban informal settlements. We combine spatial statistical methods and regression analyses to investigate differences and inequities in access to improved drinking water, improved sanitation, and consumer-reported satisfaction with water services and intermittency of water supply. We explore whether factors such as distance to water main pipes, socioeconomic status (SES), and household density influence access to improved sanitation and water services, and how water satisfaction varies within informal settlements in central Beira. Data on intra-neighborhood heterogeneity could support local service providers and stakeholders in planning efforts to expand and improve service delivery, and would support more equitable design solutions.

## Methods

### Ethics statement

The study was approved by the Mozambique National Bio-Ethics Committee for Health (Ref: 105/CNBS/20) and the Institutional Review Board of Emory University (IRB#: CR001-IRB00098584, Atlanta, GA). In addition, we obtained permissions from local authorities, namely Beira municipality and municipal district administrations from study neighborhoods included in the study. Credential letters were issued to be presented in all sub-neighborhoods and household visited. Additionally, courtesy meetings between the study team and city health department were held. Recruitment and consent of subjects took place at the households. Prior to enrollment, study staff fully explained and carried out the consent process and documented the procedure. Subjects provided written consent with a signature. In the case of illiteracy of the subject, study staff verbally summarized the material with the subject, and the participants were required to provide written consent by marking the document with a thumbprint.

To characterize water and sanitation access and satisfaction in low-income, urban neighborhoods of Beira City, we ask the following research questions: 1) What is the spatial heterogeneity of access to improved water and sanitation and satisfaction with water services within low-income neighborhoods? And 2) Does distance to water distribution mains drive access to a household connection to a piped water source or satisfaction with water services? Data is derived from a population-based survey conducted in 14 low-income areas from central Beira City. This survey is part of formative research for a parent study, titled “*Pesquisa sobre o Acesso à Água e a Saúde Infantil em Moçambique (PAASIM—*Research on Access to Water and Child Health in Mozambique)”, designed to assess the health impacts of piped water supply on young children in Beira.

### Study site.

**Fig 1.**
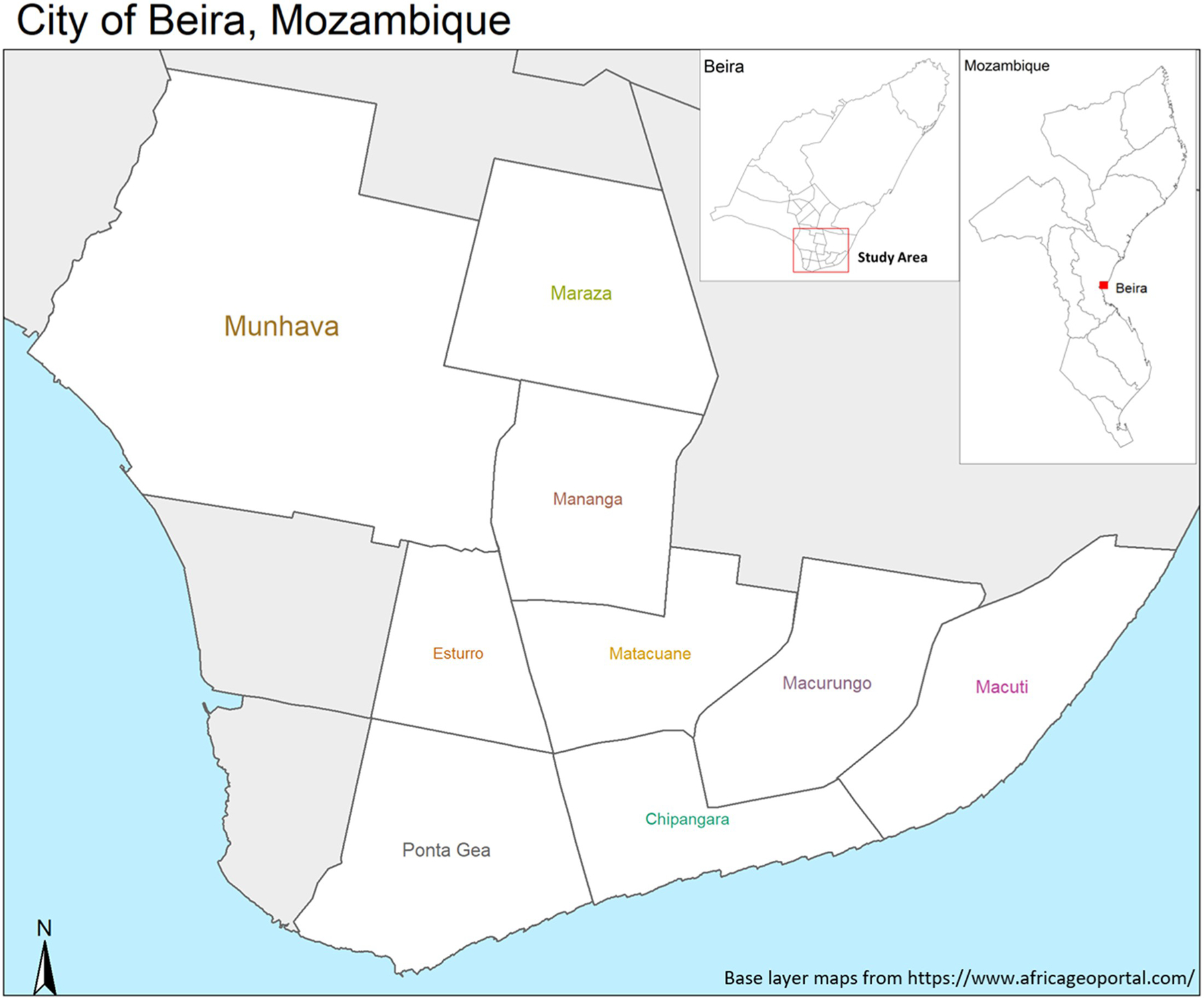
Map of the study site in Mozambique. Base layer maps were obtained from https://www.africageoportal.com, which is powered by Esri (http://www.esri.com). https://doi.org/10.1371/journal.pwat.0000022.g001

Beira, a coastal city in Sofala Province with a high-water table, at the mouth of the Púngué River, is the second-largest city in Mozambique, with a population of around 530,000 individuals (Fig 1). Mozambique’s water and sanitation sector is overseen by the National Directorate of Water Supply and Sanitation (DNAAS), Water-Supply Asset Holding and Investment Fund (FIPAG) and the Water and Sanitation Infrastructure Administration (AIAS). FIPAG is responsible for the management of assets, and both the public and private investment programs in the urban water supply systems. FIPAG is also responsible for promoting autonomous, efficient, and profitable management of the water system, namely through the transfer of operations to private operators [34, 41]. Economic regulation and consumer protection in the service provision is carried out independently from FIPAG by the Water Regulatory Authority (CRA), which as of February of 2019, became Water Regulatory Authority (AURA. IP).

To access the public distribution systems, households can open an account with FIPAG to pay for service. After the account is opened, a household connection is established if feasible. Individuals who are unable to establish or pay for a household connection can access water through public standpipes or informal arrangements with neighbors who may have a connection.

### Neighborhood selection and household sampling scheme.

We selected a set of sub-neighborhoods within the city center, primarily based on their characteristics as containing low-income (see definition in Predictor Variables), high-density, urban housing, and identified similar neighborhoods with respect to SES and population density, specifically including intervention areas that had received or were targeted to receive a new water network (intervention areas), which we are examining in the PAASIM study (Fig 1). These areas were identified and chosen using contextual information provided to us by FIPAG. Our target area was informal neighborhoods in central Beira City. This survey was conducted to validate the chosen intervention and control areas for the PAASIM study. Sub-neighborhood boundaries were delineated along natural boundaries such as roads or waterways and based on maps received from FIPAG showing areas that were scheduled to receive or not receive the intervention. We aimed to target areas inhabited by predominantly low-income residents. We then used a probability-proportionate-to-size sampling scheme to select a representative sample of single-story households (i.e., excluding multi-story apartment structures) from these areas. The occupants of these single-story households could be one family, or one person (or persons) occupying a single room with another person (or persons) occupying the other room(s).

The number of households and population density of each sub-neighborhood was approximated through household density estimates using Google Earth satellite imagery. We applied a random grid method, where a grid was placed over an area, and a random selection of squares were selected from that area. Two researchers manually counted households in the randomly selected squares, and the number of houses per unit was extrapolated across unsampled squares. Estimates of density of households in an area were used to determine proportional sampling (using household counts instead of population), where the probability of a household being selected into the study was proportional to the household density of the neighborhood. These estimates were used to get an accurate estimate for the household sampling scheme.

Enumerators used an interactive map of study neighborhoods to select a grid of their assigned segment (sub-neighborhood) to begin sampling. At this point, the enumerator randomly selected the first house using a random number generator between 1 and 19. Enumerators then systematically sampled every 19^th^ household until all households had been counted in the sub-neighborhood, to provide approximately a 5% proportional sample. The enumerator recorded sampled households that were abandoned (n = 20), had no eligible adult respondent available at the time of the survey (n = 143), or the respondent refused to consent to participate in the survey (n = 47) and moved on to the next household. The geolocation of sampled households was uploaded to the interactive map daily to ensure that areas were not missed or skipped. Community members were assigned by the sub-neighborhood head to help guide the enumerators to areas if there was no clear path. Vertical slums and two-story households were excluded from the study due to logistical challenges with sampling and conducting surveys. Further, observations during our study site selection suggested that these households were not representative of low-income urban neighborhoods.

### Data collection.

Data were collected from November to December 2019. The survey instrument consisted of several modules, including questions regarding household demographics, assets and wealth indicators, water and sanitation access, and satisfaction with water service. The survey was administered electronically on password-protected mobile tablets by enumerators. Tablets were equipped with Open Data Kit (ODK) Collect, an open source program which allows offline data collection on a mobile device [42]. A secure ODK compatible aggregation server was deployed for hosting the survey form and gathering the survey data. Submitted data were exported daily to ensure data quality (e.g., quality assurance using geocoded data to ensure households were within study area boundaries and spot checks to assess for missing survey data).

### Outcome variables.

#### Water source.

Respondents were asked to provide information on the main source of drinking water for members of the household. We classified households with a piped drinking water source located within the household or on the premises, with availability when needed, as “household connection”. Households without access to a piped water source at the household or on the premises were classified as “non-household connection”. These definitions were used to reflect criteria for safely managed drinking water according to service ladders of the Joint Monitoring Programme for Water Supply and Sanitation (JMP) [11].

#### Sanitation facilities.

Respondents were asked to provide information on the main type of sanitation their household uses and whether the main type of sanitation is shared with people outside their household. Households with a basic sanitation facility—per JMP definitions—had an unshared facility [11]. Limited or unimproved sanitation facilities include the use of pit latrines without a slab or platform and could be shared between two or more households.

#### Satisfaction with water service.

Respondents were also asked to report how often (never/sometimes/always) they are satisfied with overall service, water pressure, and water quality of their main source of drinking water. We asked respondents to report (yes/no) if there had been any time in the last month when the household did not have sufficient quantities of drinking water when needed. The responses to the water satisfaction questions related to service, sufficiency, pressure, and quality were recoded as binary, comparing those who were sometimes or always satisfied with their water provision (yes) to those who were never satisfied (no). A total satisfaction score was created by summing the individual binary scores (1 = yes/satisfied, 0 = no/unsatisfied), with the total score ranging between 0 and 4 for each household, and a higher score representing higher satisfaction.

#### Intermittency.

Respondents were asked about water service intermittency in two ways: first, how many days (0–7) in the past week was water available from their main source of water, and second, how many hours (0–24) on average was water available from their main source of water during that same timeframe.

### Predictor variables.

#### Household demographics and assets and wealth indicators.

We collected data on education of the primary caregiver, number of children under 5 years of age living in the household, and household density. Respondents answered ten standardized questions from the Simple Poverty Scorecard Poverty-Assessment Tool Mozambique, including questions on household size, materials, assets (S3 Table) [43]. Each question’s answer choices correspond with a point total, and points are summed over all ten questions into a poverty score. We use this poverty score to compare consumption of assets across different households, both using it as a continuous score and categorizing it into quartiles.

#### Distance to water main.

We calculated the distance of each respondent’s household to the water distribution main using a geocoded shapefile of the city’s water distribution system provided by FIPAG. A water main was defined by any pipe that had a diameter greater than 100 millimeters. The Euclidean distance between each survey respondent’s household and every water main pipe was calculated using the ‘sf’ package in R [44]. We then selected the minimum distance to a water main for each study participant for our analysis.

### Statistical analysis.

All statistical analyses are conducted using R statistical software (RStudio v. 1.3.1093). Bivariate analyses are conducted to describe the relationship between demographic variables and each of the outcome variables. We use unadjusted, logistic regression models to characterize associations between access to a household water connection or an unshared sanitation facility and sociodemographic variables (e.g., SES quartile) and water satisfaction responses.

We assess whether there is statistical spatial heterogeneity in water satisfaction responses and household access to water and improved sanitation facilities. We apply a kernel density estimation approach to generate a spatial relative risk surface, which describes whether the density of a specific response in space is statistically different than the density of another response. Kernel density surfaces of bivariate density are generated for responses to each question using an over-smoothing, adaptive bandwidth approach. We then use the leave-one-out least-squares cross-validation (LSCV) risk function from the ‘sparr’ package [45] to select a jointly optimal, adaptive bandwidth for the kernel density surfaces from each question and use raster algebra to create the relative risk surface which contrasts the ratio of the numerator (e.g., at least sometimes satisfied) to the denominator (e.g., never satisfied). The resulting surfaces are mapped with *p*-value contours at an alpha level of 0.05, highlighting statistical spatial density of survey responses (R-package: ‘spatstat’) [46]. All maps are generated using the ‘tmap’ package [47].

We use log-binomial regression to estimate the association between distance from water main pipe and having a household water connection. We use logistic regression to estimate the association between distance from water main pipe and satisfaction with water pressure, quality, service, and sufficiency. We assess whether there was effect modification by onsite access to an improved water source on the relationship between distance to water main and satisfaction with water pressure. We use linear regression models to estimate the association between distance from the water main and total satisfaction score. Linear regression model assumptions are checked by analysis of partial plots, residual analyses, Q-Q plots, and variance inflation factors (R-package: ‘car’); remedial measures are taken if applicable. Final models are stratified by interaction variables, when appropriate. Household density and SES score are included as confounders in each of the models based on a priori criteria.

## Results

### Water and sanitation access by sociodemographic profile

A total of 773 (47.6%) households report a household water connection (Table 1). 46% of respondents report piped water into their yard as their main source of water. 42% of respondents report piped to a neighbor as their main source of water. The remaining 12% utilize a public tap, unprotected well, borehole, bottled water, or other sources. Households in the wealthiest two SES quintiles (compared to the lowest quintile), those with a primary caregiver with a high school or above education (compared to no formal schooling), and with more than eight people living in their household (compared to 1–4 people in their household) are more likely to have access to a household water connection. Those living with one or two children under five-years-old (compared to having no children) are less likely to have access to a household water connection.

A total of 862 (54%) households report household access to a basic sanitation facility (Table 2). Households in the wealthiest socio-economic quintiles (compared to the lower quintile) and those with five or more people living in their household (compared to 1–4 people in their household) are more likely to have access to a basic sanitation facility. Those having one child under five-years-old (compared to having no children) are less likely to have access to a basic sanitation facility.

### Satisfaction with water services by household water access

For respondent-reported satisfaction with their water services, the total satisfaction mean score (0–4, with 4 being highest) is 3.45 (standard deviation (SD) 0.90) from respondents with a household water connection, and 3.44 (SD 0.83) for those without a household connection (Table 3). No differences are observed in satisfaction with water services, pressure, or quality between those with and without household water connections. We find no association between days of intermittency and a household water connection (0.01, 95% CI: −0.0,0.03), but those with a household water connection experience an increase in hours of water availability by a factor of 0.01 (95% CI: 0.01, 0.01).

### Spatial heterogeneity of household water and sanitation access and consumer-reported water satisfaction

**Fig 2.**
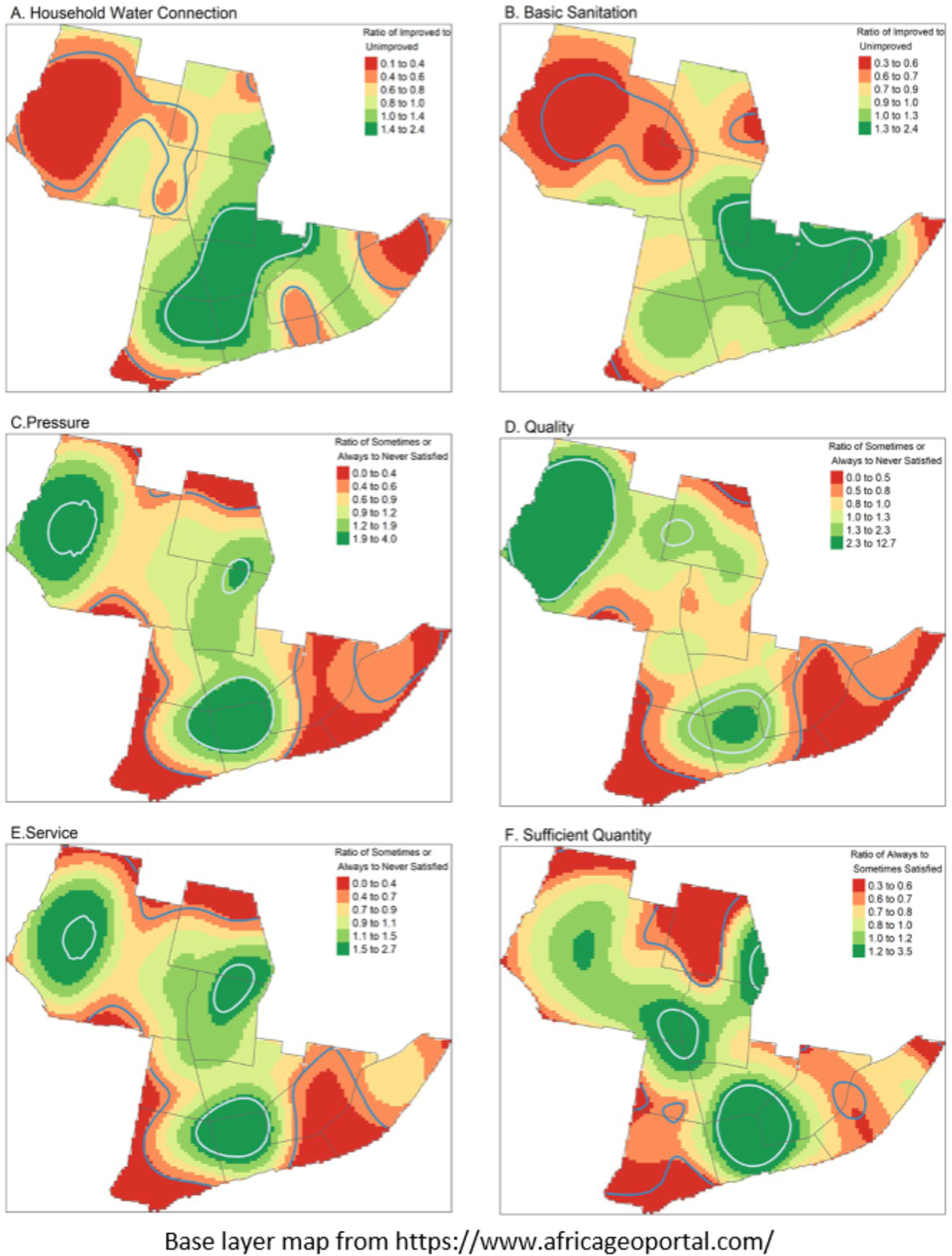
Relative risk surface of consumer-reported water satisfaction and improved water and sanitation access at the household. P-value contours in blue and white indicate areas with statistically different high or low density of survey responses. A ratio value of 1 indicates when the probability of either response at a specific location are equal. A higher ratio indicates a higher probability of having household access to improved water or unshared sanitation services or being at least sometimes satisfied with the water services. An adaptive bandwidth selection was used to select the optimum bandwidth for each individual relative risk surface. Base layer maps were obtained from https://www.africageoportal.com, which is powered by Esri (www.esri.com).

We identify statistical spatial heterogeneity in household access to water and basic sanitation (Fig 2A and 2B), as well as consumer-reported satisfaction with water services, pressure, quality, and sufficient quantity among our study participants within the city of Beira (Fig 2C–2F). The relative risk surfaces displayed in Fig 2 outline areas within the city that have higher (green) or lower (red) density of access or lack of access, and satisfaction or lack thereof. Statistically high or low density of responses at an α-level of <0.05 are indicated by the contour line colored in white and blue, respectively. Some areas contain both statistically high and low access or satisfaction.

Although the number of participants who had a household water connection and a basic sanitation facility are similar, those who have access to basic sanitation at the household are not always the same as those who have household water connections, indicated by the differences in the colored hotspots found in Fig 2A and 2B. Spatial patterns are similar across water satisfaction metrics (Fig 2C–2F), but these metrics do not always overlap with household water connections (Fig 2A).

### Association between intermittency in water availability and satisfaction with water services

Intermittency in water supply, both by days and hours, is associated with each of the satisfaction variables. Reporting having access for a greater number of days in the past week or hours in the past day is associated with an increased odds of responding as being satisfied with water quality, pressure, satisfaction, service, and sufficiency (S1 Table).

### Association of distance from water main with household access to water and satisfaction with water services

We find an inverse association between distance from water main and both access to a household water connection and satisfaction with water pressure, service, and sufficiency (i.e., as distance went up, satisfaction went down), controlling for household density and SES score. Using log-binomial regression, we find that for every 100-meter increase in distance from a water main pipe, the prevalence of household access to an onsite water source was 13% lower (OR 0.87, 95% CI: 0.82, 0.92), controlling for only household density. That is, the further a participant is from a water main, the lower the odds are that they have access to an onsite water source. The model does not converge with SES score included, so it is excluded from the model. We also compute an odds ratio using logistic regression with SES score included, and find a similar effect estimate for the association between odds of a household having access to an onsite water source (OR: 0.82, 95% CI: 0.82, 0.92). Similarly, the odds of responding ‘sometimes’ or ‘always’ satisfied with water pressure—compared to ‘never’–decrease by 20% for every 100-meter increase in distance from the closest water main pipe (OR: 0.80; 95% CI: 0.69, 0.94).

The odds of responding ‘sometimes’ or ‘always’ satisfied with water service decrease by 18% for a 100-meter increase in distance from the water main (OR: 0.82, 95% CI: 0.70, 0.95). The odds of responding ‘always’ satisfied with water sufficiency-compared to the response of ‘insufficient at least once’- decrease by 21% for every 100-meter increase in distance from the water main (OR: 0.79, 95% CI: 0.71, 0.88). There is no association between distance from water main and satisfaction with water quality (OR: 1.02, 95% CI: 0.88, 1.19). There is no effect modification by household access to water on the relationship between distance from water main and satisfaction with water pressure, quality, or sufficiency. SES and household density are confounders and subsequently included each of the models. We also observe an inverse association between total satisfaction score and distance from the water main, controlling for household density and SES score. For every 100-meter increase in distance from the water main, total satisfaction score is reduced by a factor of 0.08 (95% CI: −0.13, −0.04) (Table 4). Distance from water main is also associated with intermittency. For every 100-meter increase in distance from the water main, days of availability in the past week decreases by a factor of 0.22 (95% CI: −0.29, −0.15); hours of availability decrease by a factor of 0.30 (95% CI: −0.59, −0.01).

## Discussion

We examine the heterogeneity in access to household water connections and sanitation, and satisfaction with water services in low-income urban neighborhoods of Beira, Mozambique. By combining spatial statistical methods and regression analyses, we investigate whether locally-heterogenous factors such as distance to water mains influence access to household water and basic sanitation facilities, and how access and water satisfaction vary. We find that higher SES, higher education of the primary caregiver, and having more people but less children under five in the household are all associated with having a household water connection.

We find substantial spatial heterogeneity in access to and satisfaction with WASH services, even within a low-income, underserved area of the city. Distance to water mains is a key predictor of water access services, intermittency, and satisfaction, even over relatively short distances within neighborhoods.

In our study, we identify several demographic factors that are associated with having a household water connection. Individuals in the higher wealth quartiles (Q3 and Q4) are more likely to have a household water connection. This makes sense given that the household water supply is a paid service through FIPAG, and aligns with findings from a study of access to environmental health assets across 41 low- and middle-income countries, where individuals with higher economic wealth were more likely to have access to in-house piped water connections [48]. We also identify a positive association between having a household density of greater than 8 individuals living in the household and having a household water connection. This is potentially a function of wealth; there could be a greater number of income-generating individuals contributing to household assets which could increase the likelihood of having an onsite water connection established. Education of the primary caregiver is associated with a household water connection. In 39 low- and middle-income countries, the association between education of the primary caregiver and child growth was mediated by access to household resources such as internal water facilities [49]. Education likely serves as a proxy for other demographic characteristics that make water access more affordable for higher SES households. Indeed, in Maputo, Mozambique, individuals with household connections had higher SES than individuals who utilized public standpipes or purchased water from their neighbors [35, 37]. Lastly, the number of children under 5 in the household is associated with a household water connection; those with 1–2 children are more likely and 3 or more children were less likely, as compared to no children. As with educational status, household crowding may serve as a proxy for underlying SES.

We identify an inverse association between distance from water main pipes and access to a household water connection; the further the compound is from the water main, the less likely residents are to have onsite water access. This pattern is consistent as difference in distance increased (i.e., for a 500-meter increase in distance, odds of having onsite access to water decreased by 63%). While this result is expected given the principles of water distribution system engineering (i.e., household connections become more difficult to implement further from the distribution main), it is an important factor to consider in the infrastructure development process. Different approaches for water service delivery may be needed for those areas that are further from the water mains, particularly for those living in informal settlements [50, 51]. The increase in urbanization in Mozambique and other LMICs has resulted in a disproportionate concentration in informal settlements, resulting in challenges related to water expansion and sanitation services [24]. Inequities in access to improved water system are closely linked to poverty, and continuing water insecurity further exacerbates already rising inequalities, resulting in prolonged public health concerns such as the spread of infectious diseases, malnutrition, limited economic development, and women and girls’ labor inequities [15, 24, 52]. Understanding predictors of access to an improved water source, such as distance to the water main pipes, can provide insight into water service expansion planning, which is a critical challenge in achieving sustainable development goal target 6.1, ensuring safe water access for all, particularly in urban areas [23, 25, 53, 54].

We find a similar inverse relationship between distance from the water main and service satisfaction scores and intermittency, in addition to spatial heterogenity in satisfaction with water services. One potential explanation for this result is that as the distance between the consumer from the water main increases, the length of the pipes that supply water to that consumer also increases. Longer service lines may have more connections, more stagnation, temperature fluctuations, and lower pressure, which could result in a lower satisfaction with water quality and service, as well as issues with water supply intermittency [55, 56]. While we do not see evidence for effect modification of water pressure, quality, or sufficiency, there might not be enough variability in the binary water pressure responses to observe this effect. Water quality is not measured microbiologically in this study because our focus is on the user experience and their opinion of water quality, which affects their water consumption and management practices. The association between increased distance from water mains and more intermittent water supply highlights potential infrastructural challenges with providing water services in a rapidly urbanizing area [57]. Intermittent water supply (IWS), compared to continuous water supply, can lead to an increased risk of contamination in the water supply [58–60]. This contamination could lead to an increase in waterborne infections and cases of diarrhea among consumers [61]. Continuous water supply is critical for both access and quality. These results suggest that monitoring of water quality, pressure, intermittency, and service on the most distal parts of the water system is important in improving the overall quality of the distribution system.

While water access is increasing in Mozambique, inequity remains a concern. We find statistical spatial heterogeneity in access to a household water connection and basic sanitation facilities within low-income neighborhoods of Beira. Previous estimates from the World Bank of water and sanitation coverage in Mozambique have only been applied on a regional scale, and assessed urban-rural disparities [11, 31]. Such estimates usually describe rapid increases in WASH access in urban areas. Our results demonstrate that even city-wide estimates of water and sanitation coverage do not capture the local heterogeneity in access to these services. These findings align with previous research conducted in Maputo, Mozambique, which identified variability across neighborhoods in coverage, service provision, and reliability of service [35, 38]. In this analysis, we explore potential mechanisms for this result (e.g., distance from water main) which indicate both financial and engineering constraints that underlie inequities in WASH-access. Such findings underpin discussions that global measures of coverage are largely overestimated, and highlight how little attention urban informal settlements receive relative to surrounding urban centres when it comes to development [23, 26].

The unequal distribution of household connections to an improved water supply has important consequences. First, consumers who utilize a public standpipe tend to pay 3–4 times more for their water than those who have a private connection in their household or on their compound; these consumers face the additonal burden of time used and physical effort expended to collect their water from local sources [5]. An alternative to using a public standpipe is purchasing water from neighbors, which has been associated with lower satisfaction with water quality and a decrease in water availability [5]. Finally, water that is collected from outside of the household is subsequently stored and is subject to contamination with fecal material [37, 62, 63]. Thus, understanding how hetergeneous access to householdwater services influences behaviors around water usage is important to improve the control of water-borne diseases.

Households with water connections do not always have access to basic sanitation facilities. This phenomenon aligns with global reports of improved water access having increasing at a greater rate than access to improved sanitation facilities [64]. Historically, funding agencies have been more willing to invest in water infrastructure than in sanitation [65, 66]. This is likely due to the way that water and sanitation is defined by the WHO/UNICEF Joint Monitoring Program. “Basic” sanitation is the presence of a private (household) facility, but can include a non-sewered pit latrine. Indeed, there are few sewered connections in our target neighborhoods because sewers are not available. Another potential reason for this finding is the differences in barriers to providing access to a household water connection and improved sanitation in informal settlements. Specifically, differences in access can be driven by the location of the settlement [67]. For example, a high water table, such as in Beira, can impede the installation of sanitation facilities such as pit latrines [3, 34, 66]. Further research should be directed towards the design of interventions to navigate the specific challenges related to the structure of informal settlements. Another potential explanation for this finding is the separation of water and sanitation utilities in Beira. Sanitation services (i.e., sewers) are not provided centrally within much of the informal neighborhoods where the survey was conducted, so we would not expect that the households that have water connections also have access to basic sanitation services. Spatial maps, such as the ones we produced, could support planning and targeting of low-income and poorly served areas, recognizing the different engineering requirements of sanitation and water access.

In this study, we combine multiple analytical methods to explore neighborhood and sub-neighborhood heterogeneity of water access and satisfaction in Beira, Mozambique. Our study provides visual mapping of access to water and sanitation services to facilitate our understanding in the variability of urban coverage of access to improved water and sanitation facilities on a fine scale. Moreover, we are able to discern local heterogeneity of water access and satisfaction as a result of distance to water main. It is important to understand these heterogeneities when trying to identify the hardest to reach communities and achieve equitable access to a safe, continuous water supply. Equitable access in this context is a function of both the ability of the consumer to afford service, as well as the ability of the water utility to allocate water pressure and establish connections within informal settlements. It is not sufficient to construct a new piped water network; in these settings poorer people are often less serviced despite what appears to be a homogenous improvement in the allocation of services via the new network. Similarly, providing subsidies or other financial solutions are not sufficient if engineering constraints are the fundamental limiting factor in water service provision.

This study is also subject to limitations. We conducted the survey only in the low-income areas of Beira. Future research could measure and map city-wide access to water services and satisfaction to support more holistic and equitable planning. Survey questions related to water satisfaction were subjective and inherent to recall bias. We piloted the survey with a wider Likert scale and did not find sufficient heterogeneity in the response to warrant inclusion in the final tool. Although satisfaction is not binary, we are not able to capture those subtle differences within this analysis. Participants responses to water satisfaction questions may also be influenced by neighbors’ access to water services. Regardless, citizen reports on satisfaction are important data for city planners to collect as these are often not captured by water utilities [68]. Additionally, we did not collect an empirical data on the microbiological quality of the water or pressure measurements from the water source. Future studies could investigate the relationship between consumer satisfaction and perceived injustices or inequalities with water services. Additionally, standardized sampling methods could be applied to help avoid or limit needing recall information.

## Conclusion

Few studies have explored intra-neighborhood water and sanitation access in low-income urban neighborhoods. To our knowledge, this study is the first to utilize a spatial analysis approach to assess local water and sanitation access and satisfaction in Mozambique, and the first to assess the impact of distance from water mains and household water access and satisfaction. We find associations between household water access and household density, wealth, and education of the primary caregiver, as well as substantial spatial heterogeneity in access to and satisfaction with WASH services in low-income urban areas of Beira, even across small scales. Distance to water main is a key predictor of water access services, satisfaction, and intermittency in water services. This finding highlights the challenges of providing equitable access to water in urban informal settlements, the need for infrastructural solutions that increase safe water access and pressure throughout neighborhoods, and the development of hybrid models of water service delivery that address heterogeneity in access even in areas that are theoretically served by piped water connections. Future research could explore solutions that allow for the manageable and sustainable expansion of service coverage without sacrificing quality. Given the wealth inequities in household water connection access, exploration of solutions related to increasing the affordability of service (e.g., subsidizing the cost of establishing a connection) is also warranted. Understanding how heterogeneous access to improved water services influences water usage and behaviors can have implications for waterborne diseases, time savings, health, and well-being in the growing urban areas of the world.

## Supplementary Material

Supporting Information Table S1 S1 Table. Association between intermittency and satisfaction questions.

Supporting Information Table S2 S2 Table. Study neighborhoods.

Supporting Information Table S3 S3 Table. Standardized questions from the “Simple Poverty Scorecard Poverty-Assessment Tool Mozambique,” which included questions on household size, materials, assets.

Supporting Information - Inclusivity in Global Research S1 File. Inclusivity in global research.

Supporting Information Figure S1 S1 Fig. Distance from water main category by SES quartile.

Supporting Information Figure S2 S2 Fig. Distance to water main by satisfaction score.

## Figures and Tables

**Table 1.** Socio-demographic profile of study recruits by household water connection in Beira, Mozambique.

	Household water connection*		OR (95% CI) ^†^	*p-*value^#^
	Yes	No		
**All respondents**	773 (48%)	836 (52%)		
**Socioeconomic quartile**				
Q1 (poorest)	198 (26%)	285 (34%)		Ref.
Q2	249 (32%)	293 (35%)	1.22 (0.95,1.57)	0.11
Q3	176 (23%)	157 (19%)	1.61 (1.22, 2.14)	<0.001
Q4 (wealthiest)	150 (19%)	101 (12%)	2.14 (1.57, 2.92)	<0.001
**Education level of primary caregiver**				
No formal schooling	68 (9%)	78 (9%)		Ref.
Primary School	114 (15%)	131 (16%)	1.00 (0.66,1.51)	0.99
Secondary school	223 (29%)	335 (40%)	0.76 (0.53,1.10)	0.15
High school or above	368 (48%)	292 (35%)	1.45 (1.01, 2.07)	0.04
**Children** <**5yrs living in household**				
0	377 (49%)	323 (39%)		Ref.
1	244 (32%)	328 (39%)	0.64 (0.51,0.80)	<0.001
2	124 (16%)	150 (18%)	0.71 (0.54,0.94)	0.02
3+	28 (4%)	35 (4%)	0.69 (0.41,1.15)	0.15
**No. of people living in household**				
1–4	264 (34%)	346 (41%)		Ref.
5–7	252 (33%)	270 (32%)	1.22(0.97, 1.55)	0.09
8+	257 (33%)	220 (26%)	1.53 (1.20,1.95)	<0.001

* ’Household water connection’ was defined as those having piped water in own dwelling/yards.

† Odds ratio (OR) and 95% confidence intervals of an improved source of drinking water facilities among subjects’ demographics were compared to those without using separate logistic regression models for each characteristic.

# P-values were obtained using chi-square tests.

**Table 2.** Sociodemographic profile of study recruits by basic sanitation services in Beira, Mozambique.

	Basic sanitation*		OR (95% CI) ^†^	*p-*value^#^
	Yes	No		
**All respondents**	862 (54%)	749 (46%)		
**Socioeconomic quartile**				
**Q1 (poorest)**	240 (28%)	248 (33%)		Ref.
**Q2**	282 (33%)	262 (35%)	1.11 (0.87,1,42)	0.39
**Q3**	175 (20%)	155 (21%)	1.17 (0.88,1.54)	0.28
**Q4 (wealthiest)**	165 (19%)	84 (11%)	2.03 (1.48,2.79)	<0.001
**Education level of primary caregiver**				
**No formal schooling**	76 (9%)	71 (9%)		Ref.
**Primary School**	123 (14%)	119 (16%)	0.97 (0.64,1.46)	0.87
**Secondary school (grades 5–8)**	294 (34%)	268 (36%)	1.02 (0.71, 1.47)	0.89
**High school or above**	369 (43%)	291 (39%)	1.19 (1.83, 1.70)	0.35
**Children** <**5yrs living in household**				
**0**	390 (45%)	303 (40%)		Ref.
**1**	291 (34%)	286 (38%)	0.79 (0.63, 0.99)	0.04
**2**	141 (16%)	136 (18%)	0.81 (0.61, 1.07)	0.13
**3+**	40 (5%)	24 (3%)	1.30 (0.76, 2.20)	0.34
**No. of people living in household**				
**1–4**	234 (27%)	376 (50%)		Ref.
**5–7**	295 (34%)	229 (31%)	2.07 (1.63, 2.63)	<0.001
**8+**	333 (39%)	144 (19%)	3.72 (2.88,4.79)	<0.001

* Basic sanitation facilities were defined as improved include flush/pour flush to piped sewer systems, septic tanks or pit latrines; ventilated improved pit latrines, composting toilets or pit latrines with slabs not shared with other households. Non-basic (i.e., limited or unimproved) sanitation facilities include pit latrines without a slab or platform, hanging latrines or bucket latrines shared between two or more households.

† Odds ratio (OR) and 95% confidence intervals of having improved sanitation facilities among subjects’ demographics were compared to those without using separate logistic regression models for each characteristic.

# P-values were obtained using chi-square tests.

**Table 3.** Frequency profile of respondent-reported satisfaction with and access to water services in Beira, Mozambique.

	Household water connection			
	Yes	No		
Total satisfaction score*	Mean (sd)	Mean (sd)	β (95% CI)	*p-*value
	3.45 (0.90)	3.44 (0.83)	0.01 (−0.07,0.10)	0.75
	**N (%)**	**N(%)**	**OR (95% CI)**	***p-*value**
**All respondents**	773 (48%)	836 (52%)		
**Satisfaction**				
**Services**				
Always Satisfied	181 (23%)	145 (17%)	1.09 (0.63, 1.91)	0.76
Sometimes Satisfied	520 (67%)	617 (74%)	0.82 (0.50, 1.34)	0.43
Never Satisfied	71 (10%)	74 (9%)	Ref.	Ref.
**Pressure**				
Always Satisfied	284 (37%)	215 (26%)	1.31 (0.73, 2.33)	0.36
Sometimes Satisfied	428 (55%)	538 (64%)	0.76 (0.44, 1.32)	0.33
Never Satisfied	58 (8%)	72 (10%)	Ref.	Ref.
**Quality**				
Always Satisfied	78 (10%)	96 (11%)	0.55 (0.30, 1.01)	0.05
Sometimes Satisfied	599 (77%)	649 (78%)	0.83 (0.54, 1.28)	0.40
Never Satisfied	96 (13%)	91 (11%)	Ref.	Ref.
**Sufficient Quantity**				
Always Sufficient	577 (75%)	612 (73%)	1.08 (0.86,1.35)	0.49
Insufficient at least once	196 (25%)	224 (27%)		Ref.
**Intermittency**	**Mean (sd)**	**Mean (sd)**	**β (95% CI)**	***p-*value**
Days of access in previous week	6.3 (1.5)	6.2 (1.5)	0.01 (−0.00, 0.03)	0.15
Hours per day in previous week	11.4 (6.0)	10.1 (5.8)	0.01 (0.01, 0.01)	<0.01*

* A total satisfaction score was created by summing the individual binary scores (services, pressure, quality, and sufficient quantity), with the total score ranging between 0 and 4 for each household, and a higher score representing higher satisfaction.

† Odds ratio (OR) and 95% confidence intervals of an improved source of drinking water facilities among satisfactions were compared to those without using separate simple logistic regression models for each characteristic.

**Table 4.** Assessment of the relationship between distance from water main pipe on household water access and consumer-reported satisfaction with water. Household density and SES score are included as covariates in each of the logistic regression models. SES is not included in the log binomial model for the association between distance from water main pipe and household water connection due to failed convergence. The coefficients correspond to a 100-meter increase in distance from the water main pipes.

Outcome	Effect estimate	95% CI	*p*-value
Household water connection	PR: 0.87^#^	0.82, 0.92	<0.01
Water pressure satisfaction*	OR: 0.80^†^	0.69, 0.94	0.01
Water quality satisfaction*	OR: 1.02^†^	0.88, 1.19	0.84
Water service satisfaction*	OR: 0.82^†^	0.70, 0.95	0.01
Water sufficiency satisfaction*	OR: 0.79^†^	0.71, 0.88	<0.01
Total satisfaction score	β: −0.08^^^	−0.13, −0.04	<0.01
Intermittency (days)	β: −0.22	−0.29, −0.15	<0.01
Intermittency (hours)	β: −0.30	−0.59, −0.01	0.04

# Estimate is prevalence ratio computed using log binomial regression. This model included household density as a covariate, but not SES score.

† Estimates are odds ratios computed using logistic regression. Household density and SES score were included as covariates in each of these models.

* Comparing responses of ‘Always’ or ‘Sometimes’ satisfied to the response of ‘Never’ satisfied.

^ Estimate is the coefficient computed using linear regression. Household density and SES score were included as covariates in this model.

## Data Availability

Deidentified raw data and analysis code can be accessed on our project OSF (Open Science Framework) site upon publication at this link: https://osf.io/2a963/. Geocoded locations of study participants used to generate the maps will not be publicly available due to ethical concerns and protection of our study participants.
