# Supplementary material for: Spatial heterogeneity of neighborhood-level water and sanitation access in informal urban settlements: A cross-sectional case study in Beira, Mozambique
Source: PLOS Water. Author manuscript; Available in PMC 2022 Oct 17. (PMC9573900; doi:10.1371/journal.pwat.0000022)
Supplement: Supporting Information Table S1 — S1 Table. Association between intermittency and satisfaction questions. [file NIHMS1835935-supplement-Supporting_Information_Table_S1.docx]

S1 Table: Association between intermittency and satisfaction questions

|  | **Intermittency (days)** | | **Intermittency (hours)** | |
| --- | --- | --- | --- | --- |
| **Satisfaction** | **OR (95% CI)** ^†^ | ***p*-value** | **OR (95% CI)** | ***p*-value** |
|  |  |  |  |  |
| Quality | 1.38 (1.27, 1.49) | <0.01 | 1.04 (1.01, 1.08) | <0.01 |
| Pressure | 1.51 (1.39, 1.65) | <0.01 | 1.14 (1.09, 1.19) | <0.01 |
| Service | 1.56 (1.44, 1.70) | <0.01 | 1.10 (1.05, 1.14) | <0.01 |
| Sufficiency | 1.73 (1.59, 1.88) | <0.01 | 1.08 (1.06, 1.11) | <0.01 |
|  | **β (95% CI)^#^** | ***p-*value** | **β (95% CI)** | ***p-*value** |
| Total Satisfaction Score | 0.27 (0.24, 0.29) | <0.01 | 0.03 (0.02, 0.03) | <0.01 |

^†^Estimate is the odds ratio computed using simple logistic regression with only the predictor variable (e.g., Quality) in the model.

^#^Estimate is the coefficient computed using simple linear regression with only Total Satisfaction Score in the model.
