# Supplementary material for: Spatial heterogeneity of neighborhood-level water and sanitation access in informal urban settlements: A cross-sectional case study in Beira, Mozambique
Source: PLOS Water. Author manuscript; Available in PMC 2022 Oct 17. (PMC9573900; doi:10.1371/journal.pwat.0000022)
Supplement: Supporting Information Table S2 — S2 Table. Study neighborhoods. [file NIHMS1835935-supplement-Supporting_Information_Table_S2.docx]

S2 Table: Study neighborhoods

| Macuti North |
| --- |
| East of Mananga |
| North Munhava |
| North Munhava* |
| South of Mananga |
| Esturro |
| Engen Beria |
| Macuti South |
| Matacuane |
| Maraza |
| Macarungo |
| Munhava/Mananga |
| Chipangara |
| Ponta Gea |

*Additional control area for the survey was added during data collection.
