# Supplementary material for: Spatial heterogeneity of neighborhood-level water and sanitation access in informal urban settlements: A cross-sectional case study in Beira, Mozambique
Source: PLOS Water. Author manuscript; Available in PMC 2022 Oct 17. (PMC9573900; doi:10.1371/journal.pwat.0000022)
Supplement: Supporting Information Table S3 — S3 Table. Standardized questions from the “Simple Poverty Scorecard Poverty-Assessment Tool Mozambique,” which included questions on household size, materials, assets. [file NIHMS1835935-supplement-Supporting_Information_Table_S3.docx]

S3 Table: Standardized questions from the “Simple Poverty Scorecard Poverty-Assessment Tool Mozambique,” which included questions on household size, materials, assets.

| **HOUSEHOLD ASSETS & WEALTH INDICATORS** |
| --- |
| How many bedrooms are in the house? |
| How many beds does this household have (single, double, bunkbeds, or for children)? |
| **Does your household/house have any of the following?** |
| A functioning bicycle |
| A functioning motorcycle |
| A functioning car/truck |
| Non-electric or electric clothes iron |
| Functioning freezer |
| A clock (mobile phone, wall, wrist, or pocket) |
| A radio, stereo system, or cassette player |
| A sofa |
| Grated windows |
| Does the house have a grated door? |
|  |
| What is the main source of energy for lighting in the residence? |
| Where is the kitchen located? |
| What is the main material of the floor of the residence (excluding kitchen and bathrooms)? |
| What is the main material of the walls of the residence? |
