# Supplementary figures and images for: Spatial heterogeneity of neighborhood-level water and sanitation access in informal urban settlements: A cross-sectional case study in Beira, Mozambique
Source: PLOS Water. Author manuscript; Available in PMC 2022 Oct 17. (PMC9573900; doi:10.1371/journal.pwat.0000022)

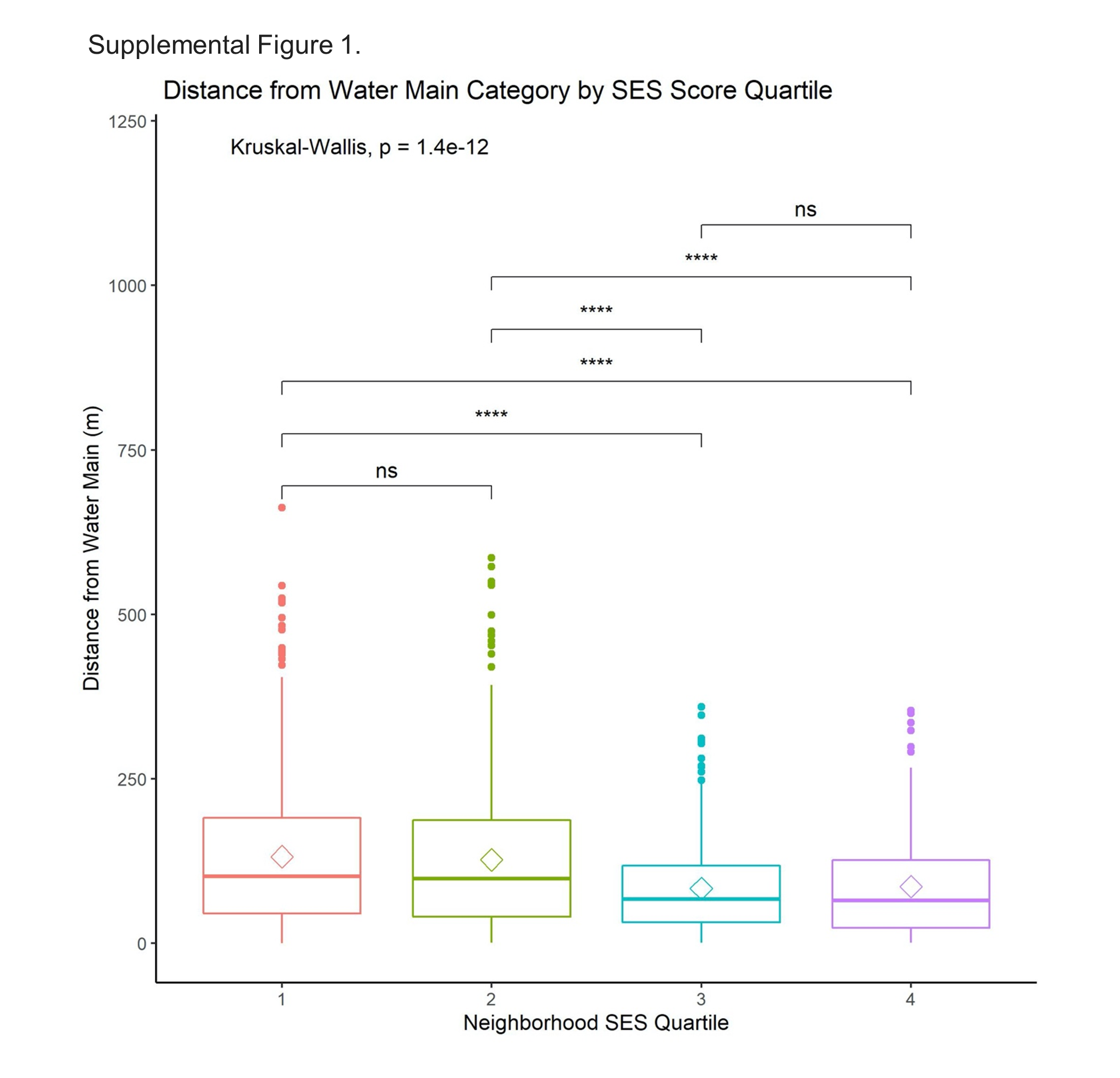

Supplement: Supporting Information Figure S1 — S1 Fig. Distance from water main category by SES quartile. [file NIHMS1835935-supplement-Supporting_Information_Figure_S1.tif]

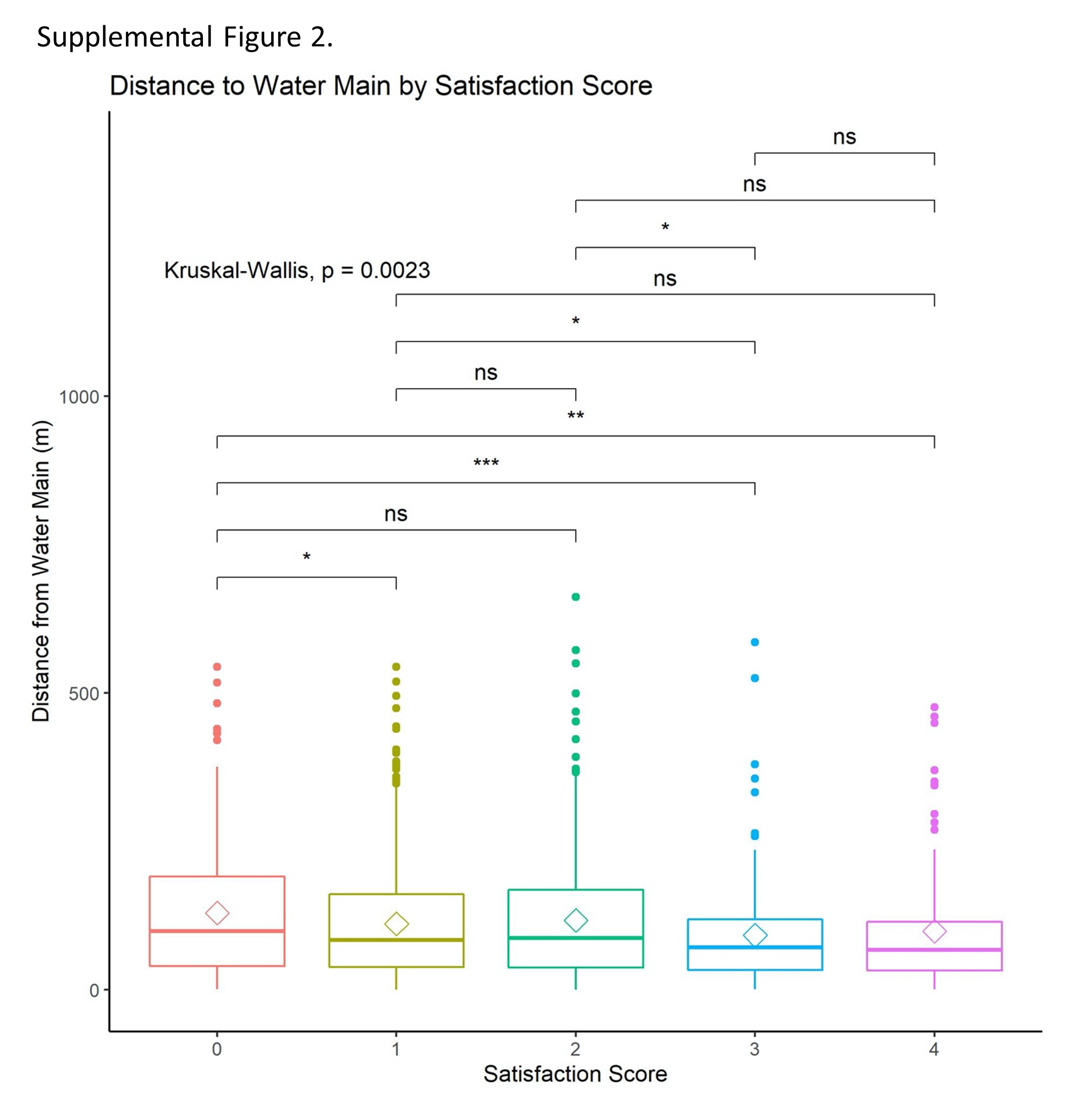

Supplement: Supporting Information Figure S2 — S2 Fig. Distance to water main by satisfaction score. [file NIHMS1835935-supplement-Supporting_Information_Figure_S2.tif]
